# Test your knowledge and understanding

**Published:** 2013

This page is designed to test your understanding of the concepts covered in this issue and to give you an opportunity to reflect on what you have learnt. The multiple true/false questions were produced in collaboration with the International Council of Ophthalmology (ICO) and the Diagnose This quiz is provided courtesy of the Ophthalmic News and Education (ONE®) Network of the American Academy of Ophthalmology.

**Table:** 

**1.**	**Think about your understanding of disability and impairment**	**True**	**False**
a	People with impairments face barriers such as small print they cannot read, or stairs they cannot climb	□	□
b	It is helpful to show pity for people with impairments	□	□
c	The words ‘disability’ and ‘impairment’ mean more or less the same thing	□	□
d	It is better to talk to the person supporting someone who is visually impaired, rather than the person themself	□	□
**2.**	**Think about how to be more inclusive of people with impairments**	**true**	**False**
a	When you update your facilities, involving architects, eye care specialists and disabled people's organisations is the best way to get a ‘Design for All’ result	□	□
b	One of your colleagues thinks that women with disabilities have committed sins and are evil. This attitude negatively impacts on their work, but nothing can be done about it	□	□
c	The best way to find out how accessible your eye service is for people with impairments is to ask users with impairments, for example with a ‘knowledge, attitudes, practices’ (KAP) survey	□	□
d	Regularly inviting representatives from disabled people's organisations into your hospital for news/advice will help your patients receive the most effective and inclusive services	□	□
**3.**	**Think about the needs of people with different types of impairments**	**True**	**False**
a	It is best to find out what a patient with a hearing, mobility, or intellectual impairment may need before they arrive at the clinic	□	□
b	You have several medical issues to discuss with your patient. They nod in agreement with all your suggestions. You can safely assume that they have understood everything you have explained	□	□
c	You guided a patient with a visual impairment by pulling them along with you and then leaving them in the centre of the next waiting area. There are better ways you could have handled this	□	□
d	Patients need both verbal (spoken) and written instructions to ensure they take the correct drugs at the correct time once they leave the clinic	□	□

## ANSWERS

1. **1a. True. b. False**. People with impairments do not like to be pitied. It is better to treat them with the same respect and dignity you would show to people without impairments, **c. False**. Impairments refer to the physical or intellectual problem, such as hearing, visual, intellectual, or mobility impairments. People with impairments are disabled when society does not include them by making services or goods accessible to them. **d. False**. Always talk to the patient directly, no matter what impairment they have, or who else is in the room.
2. **2a. True**. Involve as many as possible of the groups affected, especially people with impairments. This will bring you closer to full inclusion, **b. False**. Training about disability discrimination may help; however, it will take more than one training session to challenge entrenched beliefs and core discriminatory attitudes, **c. True**. See Comm Eye Health J 2012; 25(78):22–24. **d. True**. Remember, people with actual experience of disability are the experts!
3. **3a. True**. This can be done by telephone, mail, or via an outreach health worker. Doing so will help both you and the patient to prepare for the visit, for example by arranging a sign language interpreter, **b. False**. It may be that the patient has a hearing impairment or a learning difficulty, or simply that they are not confident to ask questions or challenge you. **c. True**. Offer your arm so they can hold on to it while you walk. Explain what you are doing and where you are, and guide their hand to the back of a chair if they have to sit down and wait. **d. True**.

