# Supplementary material for: Test your knowledge and understanding
Source: Community Eye Health. 2013;26(81):19. (PMC3678317)
Supplement: Related texts

# Brief report: Diagnose This quiz

**Published:** 2013

A patient presents with a cataract and lenticular changes as shown. Which of the following is the most likely cause of his cataract?

- □ **Atopy**
- □ **Electrical injury**
- □ **Myotonic dystrophy**
- □ **Chronic use of topical steroids**

## ANSWER

**The most likely cause is electrical injury**

Electrical injuries, such as those associated with high-voltage electrocution, cause coagulation of lens protein and give the typical cortical-spoking lenticular changes displayed in the figure.

Approximately 90percnt; of all patients with myotonic dystrophty develop cataracts. In the early stages, the cataract presents as a diffuse opacification of the posterior subcapsular region. Later, the opacification becomes dense and often produces a stel late lesion at the posterior pole. Patients using cortico-steroids for chronic conditions commonly present with posterior subcapsular cataracts. Patients with atopy generally exhibit anterior or posterior capsular cataracts, often with stellate opacification.
